# Supplementary material for: Causal Relationship between Aging and Anorexia Nervosa: A White-Matter-Microstructure-Mediated Mendelian Randomization Analysis
Source: Biomedicines. 2024 Aug 16;12(8):1874. doi: 10.3390/biomedicines12081874 (PMC11351342; doi:10.3390/biomedicines12081874)
Supplement: Supplementary file 1 [file biomedicines-12-01874-s001.zip › Supplementary Figure/Figure S4/Singe SNP plot of MR analysis for TL to ubm-b-1531.pdf]

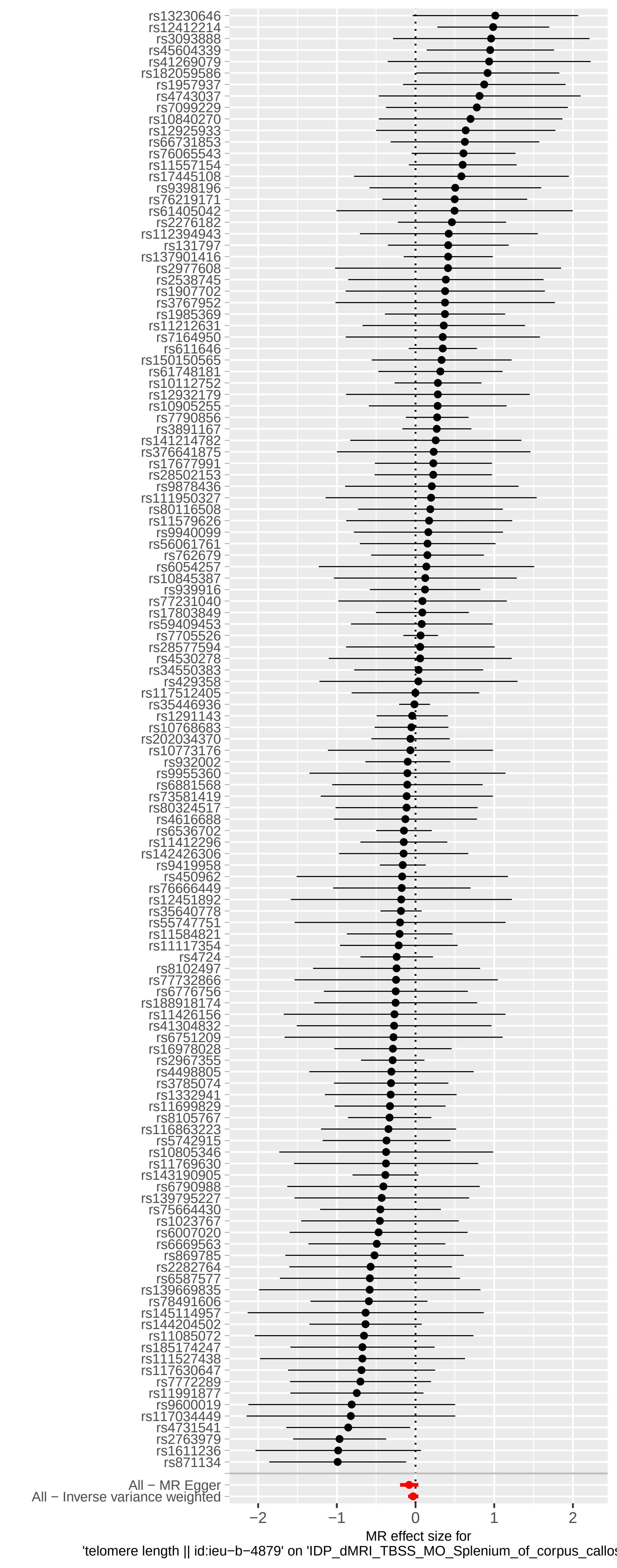
rs13230646
rs12412214
rs3093888
rs45604339
rs41269079
rs182059586
rs1957937
rs4743037
rs7099229
rs10840270
rs12925933
rs66731853
rs76065543
rs11557154
rs17445108
rs9398196
rs76219171
rs61405042
rs2276182
rs112394943
rs131797
rs137901416
rs2977608
rs2538745
rs1907702
rs3767952
rs1985369
rs11212631
rs7164950
rs611646
rs150150565
rs61748181
rs10112752
rs12932179
rs10905255
rs7790856
rs3891167
rs141214782
rs376641875
rs17677991
rs28502153
rs9878436
rs111950327
rs80116508
rs11579626
rs9940099
rs56061761
rs762679
rs6054257
rs10845387
rs939916
rs77231040
rs17803849
rs59409453
rs7705526
rs28577594
rs4530278
rs34550383
rs429358
rs117512405
rs35446936
rs1291143
rs10768683
rs202034370
rs10773176
rs932002
rs9955360
rs6881568
rs73581419
rs80324517
rs4616688
rs6536702
rs11412296
rs142426306
rs9419958
rs450962
rs76666449
rs12451892
rs35640778
rs55747751
rs11584821
rs11117354
rs4724
rs8102497
rs77732866
rs6776756
rs188918174
rs11426156
rs41304832
rs6751209
rs16978028
rs2967355
rs4498805
rs3785074
rs1332941
rs11699829
rs8105767
rs116863223
rs5742915
rs10805346
rs11769630
rs143190905
rs6790988
rs139795227
rs75664430
rs1023767
rs6007020
rs6669563
rs869785
rs2282764
rs6587577
rs139669835
rs78491606
rs145114957
rs144204502
rs11085072
rs185174247
rs111527438
rs117630647
rs7772289
rs11991877
rs9600019
rs117034449
rs4731541
rs2763979
rs1611236
rs871134
All − MR Egger
All − Inverse variance weighted
−2
−1
0
1
2
MR effect size for
'telomere length || id:ieu−b−4879' on 'IDP_dMRI_TBSS_MO_Splenium_of_corpus_callos
